# A versatile low-cost data acquisition system for small rocket engine test bench

**DOI:** 10.1016/j.ohx.2025.e00686

**Published:** 2025-08-16

**Authors:** Nathan Andreani Netzel, Daniel Strufaldi Batista, Francisco Granziera Jr., Marcelo Carvalho Tosin

**Affiliations:** State University of Londrina, Department of Electrical Engineering, Rod. Celso Garcia Cid– PR-445, 86057-970, Londrina, PR, Brazil

**Keywords:** Data acquisition system (DAQ), Rocket engine, Static firing test, Load cell, Pressure sensing, System calibration

## Abstract

Small sounding rockets often carry scientific instruments to collect data in atmospheric environments. In the context of university-level education and research, their development offers students a hands-on, multidisciplinary platform to study propulsion, aerodynamics, electronic instrumentation, and others. A critical component of sounding rocket experimentation involves engine testing and validation, which typically requires the design and implementation of a dedicated test bench. These test benches are essential for ensuring experimental safety and reliability while enabling the acquisition of accurate performance data. This work presents the design and implementation of a versatile, low-cost data acquisition (DAQ) system specifically developed for a small rocket engine test bench. The system can measure thrust and pressure by interfacing with load cell sensors and pressure transducers. The approach also prioritizes modularity, allowing future expansion or adaptation to different engine configurations. It also balances affordability and functionality compared to commercial DAQs by leveraging cost-effective hardware and software. Experimental testing has demonstrated the system’s ability to deliver accurate and reliable measurements, with noise levels comparable to those of commercial counterparts. These results indicate the system can enable students and researchers to conduct experiments effectively and ensure safety.

## Graphical abstract

**Figure:**
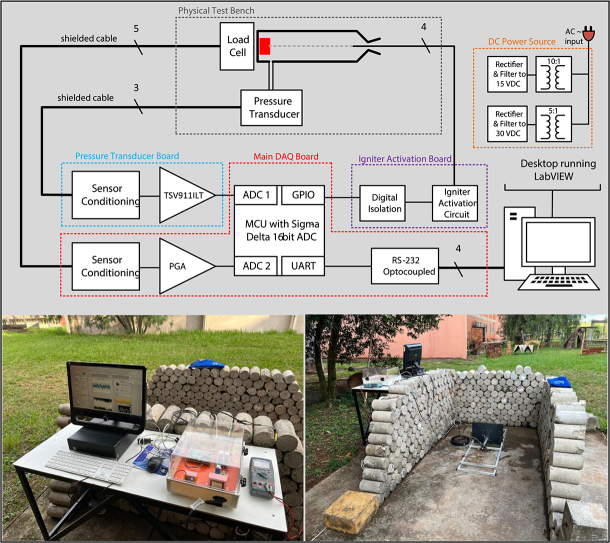

## Specifications table

**Table:** 

Hardware name	DAQ for a University Rocket Engine Test Bench
Subject area	Educational tools and open source alternatives to existing infrastructure
Hardware type	Field measurements and sensors

Closest commercial analog	The submitted hardware was developed to replace an NI USB-6221 multifunction DAQ and an amplification module for Wheatstone bridges. Nonetheless, it can replace most commercial data acquisition systems.

Open source license	Creative Commons Attribution-NonCommercial 4.0 (CC BY-NC 4.0) license
Cost of hardware	$76.34 USD (excluding the sensors)
Source file repository	https://doi.org/10.17632/c2w7759sd3.2

## 1 Hardware in context

Sounding rockets serve as platforms for carrying embedded instrumentation and collecting scientific data during atmospheric and suborbital flight missions. These missions and their data are meaningful in distinct research areas [1], [2], [3]. Moreover, the development of sounding rockets also has an important role in the research environment of worldwide universities and with educational projects in aerospace engineering and related areas [4], [5], [6], [7], [8], [9], [10], [11], [12].

These university activities or programs aim to develop functional small rockets, including work in the areas of aerodynamics, structures, vibration and aeroelasticity, propellants, engine components and systems, telemetry and telecommand, inertial systems, control, recovery systems, and others. However, due to the high complexity of aerospace technology, it is essential to perform numerous tests before flight to validate system operation and components’ integrity. These tests often involve test bench and infrastructure explicitly tailored to test and validate individual parts of the rocket or a combination of some.

One major area related to these projects is the validation and tests with the rocket engine and the propellant. Rocket design heavily relies on understanding its time-thrust profile. Ground tests are necessary for studying how changes in nozzle design, fuel mixtures, additives, and other engine factors that affect performance [10], [11], [13]. Measuring thrust during these tests offers a direct way to compare different engines and propellants. In this context, test benches are widely adopted in static rocket engine experiments to acquire data to characterize performance and validate the design parameters [4], [10], [13], [14], [15], [16], [17], [18], [19], [20], [21], [22], [23], [24].

These engine test benches for static firing tests are an infrastructure that provides a static manner to ignite and burn the engine’s propellant in a safe environment. More importantly, they have an instrumentation setup to collect vital data to assess the engine and propellant design. Usually, thrust is the most crucial information sought. Other data, such as the engine’s internal pressure, temperature, vibration, etc., might be valuable. Depending on the fuel type, there will be additional requirements, such as when liquid fuel is used. From the acquisition and subsequent processing of experimental data, it is possible to determine fundamental parameters for rocket engine research, such as thrust, specific impulse, burn time, combustion efficiency, etc.

However, although there are examples of those test facilities and setups in the literature, most barely discuss the specifics of their electronics and instrumentation behind the measurement system. For instance, using as an example the thrust measurement, most depict an overview of the test bench composition, the load cell type, its amplifying instrumentation, and the data acquisition system (DAQ) rather than describing to the reader any further details about the electronics or the DAQ operation [4], [13], [14], [17], [18], [19], [20], [25]. Primarily, those works aim to depict the test bench and its results or specific results obtained by some engine test. Instead, our work focuses on a detailed description of the test bench hardware and software design, development, operation, and validation of a solid propellant rocket engine test bench.

Furthermore, many systems are built around commercial strain gauge amplifiers and DAQs [4], [14], [17], [18], [19], [21], and, although some show a custom-built solution, the instrumentation is merely explained through diagram blocks without any details on the implementation [16], [22], [23]. Besides, some customized solutions are based on the integration of modules and development boards instead of a custom-build design [22], [23].

In this context, this work contributes by depicting the design of a low-cost data acquisition system for the test bench of a small solid propellant rocket engine. The test bench was built around a custom-designed platform incorporating commercial off-the-shelf components and custom-printed circuit boards, which were specifically tailored to the problem rather than relying on commercial electronic modules or DAQs. Compared to commercial DAQ systems and amplification modules, the proposed design is significantly more cost-effective and better tailored to the specific requirements of rocket engine testing for a laboratory environment dedicated to research and education. By being built around the sensors, the hardware avoids the need for adaptations or complementary components often required by commercial solutions. Additionally, the system incorporates enhanced safety features, including protection against voltage spikes and short circuits, to safeguard the electronics in the event of sensor or engine failures. With that, it offers a level of robustness not typically found in either commercial or generic custom-built alternatives. Similarly, authors in various fields have demonstrated the benefits of assembling custom-built test benches and DAQs [26], [27], [28], [29].

Moreover, since it is tailored to the problem, the system has a friendly user interface to perform an engine test, which allows users to focus on safety measures and ensure the security of the students and researchers involved. The resulting design maintains the expandability of a commercial DAQ, allowing a much more cost-effective solution without compromising safety and core functionality.

Nonetheless, developing such a custom system and DAQ requires knowledge of electronics and embedded systems and involves development and validation time, which are downsides compared to using a commercial system. However, the current landscape of embedded electronics has been transformed by the widespread availability of complex integrated circuits, including microcontrollers, sensors, analog front end, and amplifiers, all offering high integration density and advanced features. The accessibility of these components, combined with the availability of electronic design and simulation tools — such as integrated development environments (IDEs), electronic CAD software, and open-source libraries — as well as the services provided by companies specialized in rapid prototyping and PCB assembly, has enabled the fast and cost-effective development of dedicated electronic systems. Thus, unless time is a sensitive matter, it is unlikely that component and manufacturing costs could negate the benefit of building custom DAQ-based systems regardless of the application. Notably, authors in various fields have demonstrated the benefits of assembling custom-built test benches and DAQs [26], [27], [28], [29]. Moreover, commercial data acquisition systems typically rely on general-purpose architectures composed of various modules and subsystems that can be interconnected to serve a wide range of applications and operating environments, including industrial settings. Consequently, they come at a significantly higher cost.

The DAQ shown in this work was built as part of the Vector II project [11], [12]. This project, executed in the Department of Electrical Engineering at the State University of Londrina, Brazil, aims to build and launch a small-sounding rocket using a solid propellant engine in partnership with a local company to incentivize students in aerospace and experimental activities. At first, the engine test bench was designed using a commercial amplification module (JY-S60) with a National Instruments DAQ (NI USB-6221 DAQ).

The DAQ system was developed using an STM32F3 series ARM-based microcontroller from STMicroelectronics, featuring integrated 16-bit Sigma-Delta analog-to-digital converters. A programmable gain amplifier for bridge sensors is incorporated into the design to handle the signal conditioning of load cell sensors. The prototype also supports a pressure transducer, has isolated serial communication with a graphical user interface, and has an igniter activation module. Furthermore, its custom design focuses on expandability to allow new features in the future. The system has three distinct printed circuit boards (PCB): the main one, which contains the load cell instrumentation, the DAQ circuit, and the communication interface; the pressure transducer electronics in a second PCB; and the igniter activation PCB system. Additionally, it has all power supply systems. All of them are assessed and discussed throughout the work. The validation of the hardware depicts its noise evaluation and comparison with the commercial alternative, a calibration procedure, and the results of an actual engine test made using the system.

## 2 Hardware description

In general, the hardware is a data acquisition system for a university rocket engine test bench. Thus, the fundamental hardware criteria were established based on typical rocket engine test bench instrumentation requirements aimed primarily at university rocket designs. Accordingly, the proposed hardware matches the necessities of the Vector II Project, carried out at the State University of Londrina, and similar applications by other university research programs and experimental rocket teams. The main requirements are the following.

1. Load cell signal conditioning for a wide range operation;
2. Pressure transducer conditioning and signal amplifying;
3. Availability of Multiple ADC channels for scalability and modularity, if required;
4. Commercial off-the-shelf (COTS) integrated circuits;
5. Having a didactic and easily usable interface, allowing low complexity when performing tests;
6. Isolated serial interface via RS-232 signaling;
7. Protection circuits to prevent hardware damage in case of accidents during testing;
8. Igniter Activation circuit with remote control capabilities;
9. Expandable configuration for sensors and future integrations of external hardware to the main hardware;
10. Graphical User Interface;
11. Calibration and performance analysis.

**Fig. 1:**
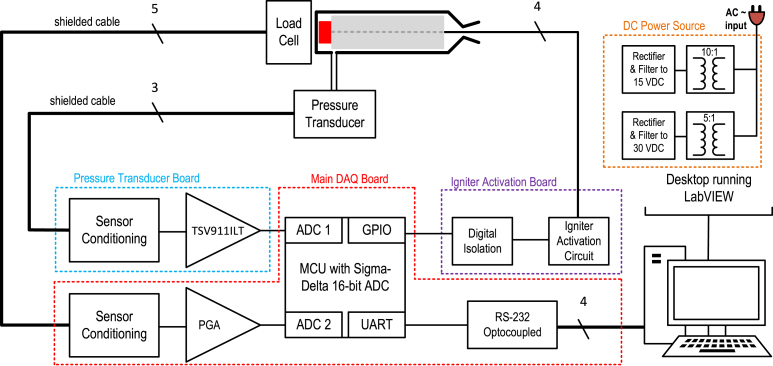
Simplified functional diagram of the data acquisition system and the engine test bench sensors.

Fig. 1 presents a diagram compiling the functional design of the data acquisition system and its sensors. The hardware consists of three modules in separate printed circuit boards: the main DAQ board, with the microcontroller, the analog-to-digital converter (ADC), the serial interface with the user interface and the load cell instrumentation circuit; the pressure transducer instrumentation, in the secondary board; and the igniter activation circuitry, in the last board. The electronic system is divided into three printed circuit boards to increase safety, modularity, and expandability. Technically, a single hardware could integrate all of the above at the expense of the system’s modularity and expandability. The following paragraphs briefly discuss the specifics of each item.

One of the most important features of the hardware is the ADC, which converts the analog output of the load cell amplifier and the pressure sensor transducer. Considering the above criteria, a viable solution is to utilize a microcontroller with an integrated Sigma-Delta ADC. Hence, the STM32F373VC microcontroller from STMicroelectronics [30] was chosen due to its three independent 16-bit Sigma-Delta A/D converters, each one with its own internal gain amplifier, which adds flexibility to the system and a wider dynamic range especially when combined with the load cell analog front end chosen for this project and described ahead. These converters support flexible input configurations, with up to 5 differential input pairs or 9 single-ended channels each, resulting in a total of up to 15 differential or 27 single-ended inputs across the three Sigma-Delta ADCs, depending on the selected configuration. The converters provide high-performance data throughput, with input sampling rates of up to 50 ksps for single-channel operation and around 16.6 ksps when multiplexing between multiple channels.

Compared to the National Instruments USB-6221, previously employed in the Vector II Project, which offers 8 differential or 16 single-ended 16-bit analog inputs with a maximum sampling rate of 250 ksps, the STM32F373VC system provides more analog input channels but with a lower maximum sampling rate per channel. Therefore, the designed system is well-suited for low-frequency, precise measurements typical in rocket propulsion testing but may be less appropriate for high-frequency applications. Furthermore, choosing a device with integrated ADCs not only reduces overall cost compared to discrete Sigma-Delta ADCs with separate controllers but also simplifies hardware design.

Additionally, the microcontroller features a 12-bit successive approximation (SAR) ADC with up to 15 input channels, offering a fast conversion time of approximately 1μs at 56 MHz, making it suitable for auxiliary measurements or general-purpose analog sensing. For digital interface, the STM32F373VC has 84 general-purpose I/O (GPIO) pins that can be used as digital inputs, outputs, or alternate functions (e.g., timers, UART, SPI, I${}^{2}$C, I^2^S). All these analog and digital interfaces make the STM32F373VC a viable microcontroller for use as the main component of any DAQ system. They can provide auxiliary measurements or general-purpose analog sensing, as well as a vast array of interfaces with sensors, actuators, and communication interfaces, without altering the core design of the Sigma-Delta ADCs used in the main analog.

Another sensitive feature is the load cell subsystem, which measures thrust during engine tests. A straightforward and effective approach is to employ a COTS component that integrates both signal conditioning and amplification. In other words, a single device capable of handling the sensor’s analog output while offering programmable gain and offset control, all managed via the microcontroller’s embedded software. This integrated approach simplifies hardware design, enhances versatility, and ensures accurate force measurement across a range of test conditions. These characteristics are essential for a reliable product and facilitate end-user operation by allowing electrical characteristics to be adjusted in software, without requiring physical modifications.

Among the available options, the PGA308, a programmable gain amplifier designed for bridge sensors [31], was selected due to its unique combination of protection features and configurability. It features three independent gain stages, allowing for a wide programmable gain range from 2.66 to 9600, and offers precise offset adjustment. Although it lacks built-in temperature compensation and internal circuitry for load cell excitation, it compensates by offering enhanced fault detection and protection capabilities.

Unlike other alternatives, such as the MAX1452 or MAX1454, the PGA308 includes several integrated safety mechanisms. One advantage of the PGA308 is the ability to detect and report numerous errors via its serial interface, alerting the user when electrical parameters exceed safe operating limits, thereby helping prevent component damage. Additionally, the device’s voltage-limiting feature is capable of limiting the amplified output voltage, regardless of any event that could lead to a voltage spike. Consequently, it provides an additional layer of protection to prevent a signal from exceeding the safe limits of the A/D converter. This feature is essential for rocket engine tests, such as the Vector II project, which previously had a commercial National Instruments DAQ damaged by a higher-than-specified voltage to the acquisition system input caused by a crushed load cell due to a motor explosion.

The instrument — specifically the main DAQ board — should be connected to a computer for integration with a proposed graphical user interface, such as a LabVIEW virtual instrument. Given that the distance between the instrument and the computer is not longer than a few meters, serial communication using the RS-232 standard is sufficient. For longer distances, a commercial RS-422 converter could be used at both ends. Furthermore, the data acquisition system should be isolated, whether galvanic or optic, from the external equipment. The isolation is required to maximize safety, to offer protection in case of accidents during testing, and to reduce noise influence in the analog instrumentation. There are many options viable, such as using RS-232 isolated integrated circuits to perform the Transistor–Transistor Logic (TTL) conversion, or typical RS-232 circuits with the isolation provided by a secondary chip making the isolation at TTL level.

In addition to the load cell measurements, the system monitors the engine’s internal pressure during testing. The pressure is acquired by an industrial transducer with a typical current output of 4–20 mA used in industrial instrumentation, which enables the transmission over distance. Furthermore, these sensors can measure high combustion chamber pressures while connected to a refrigerated copper coiled tubing, which lowers the temperature of the combustion gases. As a design decision, the electronic instrumentation of this sensor has a separate printed circuit board with extra protection against overvoltage. A current-to-voltage conversion circuit, followed by a TSV911ILT operational amplifier, is responsible for converting the output current of the industrial sensor into an analog signal and protecting the circuit against overvoltage or surges. This analog signal is then connected to one of the Sigma-Delta A/D converters in the main data acquisition board. Additional discussions are made later.

Lastly, there is the relay-operated igniter activation system. For safety reasons, the relay shorts out both terminals of the igniter to the ground when not energized, preventing an inadvertent activation due to any current flowing through the igniter’s thermal initiator. The relay must be energized to set off the igniter during actual engine testing. Also, for safety reasons, the igniter circuitry has a separate hardware and power supply. A transistor is responsible for powering the relay’s coil, and the digital signal that acts on the transistor is electrically isolated from the main DAQ microcontroller’s digital signal by an optocoupler. This signal is responsible for activating the relay when the Virtual Instrument sends the proper command to the microcontroller. Thus, although the igniter circuit is not part of the main DAQ hardware — similarly to the pressure sensor transducer conditioning — it is part of the hardware solution. The ignition board has two identical circuits that can independently drive two igniters.

The instrument must have a power supply sub-system. The main DAQ board and the Pressure Transducer Board are powered by a voltage between 13 V and 36 V, which can be provided either by a battery pack or a transformer followed by a capacitor-filtered full-wave rectifier connected to the AC power line. The igniter activation hardware is powered by a similar distinct transformer–rectifier circuit fed by the AC line or a distinct battery pack, which must generate more than 30 V to be able to initiate different types of igniters.

For a better understanding of each individual hardware, the next couple of subsections explain each of the printed circuit boards and bring a more detailed diagram in comparison to Fig. 1 overview. The schematics and more specific discussions on the components and circuits are left for the Building Instructions in Section 5.

### 2.1 Main DAQ board description

**Fig. 2:**
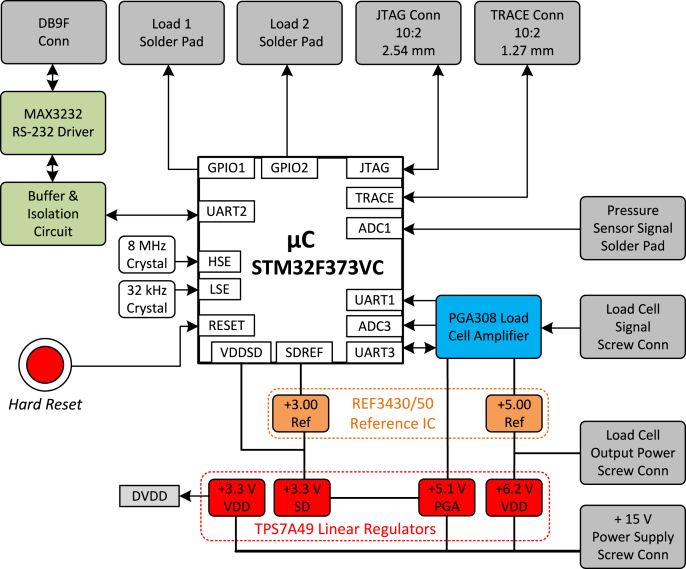
Diagram of main DAQ board.

Fig. 2 presents a detailed description of the main DAQ Board. It summarizes the main components of that hardware related to the microcontroller peripherals.

There are four linear regulators in the main DAQ board circuit. These regulators are the TPS7A49DGNT from Texas Instruments [32]. The TPS7A49 is an adjustable ultra-low noise, high Power Supply Rejection Ratio (PSRR), and low dropout voltage device, making it optimal for high-accuracy and high-precision instrumentation applications. The first regulator is powered by its external DC voltage supply. Its output is set to 6.2 V and supplies the load cell, as well as a REF3450 that generates a 5 V reference for the PGA308 internal analog circuits. Powering the load cell directly from the adjustable regulator ensures that the sensor operates at appropriate voltage levels as long as it remains above the supply required by the 5 V reference. Additionally, the regulator’s higher current capacity allows for compatibility with a wide range of load cells. A second regulator powers the PGA itself with 5.1 V. A third TPS7A49 supplies a 3.3 V to the microcontroller’s analog front end, and a REF3430 voltage reference integrated circuit is used to provide a 3.00 V to the Sigma-Delta ADC reference. This regulator is fed by the 5.1 V output of the second regulator. The last linear regulator, powered by the external supply, in the main DAQ circuit powers the remainder of the digital circuitry (3.3 V). A summary of the above description is presented in Fig. 9.

As shown in Fig. 2, the Load Cell signal is connected to a programmable analog sensor signal conditioner, the PGA308. It features three programmable gain stages, configured and calibrated via a one-wire interface connected to the UART3 of the microcontroller, which are used to finely adjust the output signal within a 0 V to 3 V range, optimizing the signal for the dynamic input range of the microcontroller’s 16-bit Sigma-Delta ADC. It also features a dual-purpose Dout/Vclamp pin, which is connected to the UART1 input pin of the microcontroller and can optionally be used to verify DOUT as digital output functionality. In our system, this pin is tied to 3.3 V, which sets the maximum output voltage to this level, protecting the microcontroller and its analog front end from an eventual load cell over-voltage output.

The signal from the pressure transducer board is routed to Sigma-Delta ADC1 of the microcontroller via a direct wired connection between the boards. The ignition command signals are routed from two distinct GPIO ports of the microcontroller and are made available at the Load 1 and Load 2 solder pads on the main DAQ board, as illustrated in Fig. 2. From these pads, the signals are directly wired to the ignition board.

A female DB9 connector on the PCB is used to establish an RS-232 serial connection with the host computer. Due to availability, the RS-232 driver used is the MAX3232E IC from Texas Instruments, a dual-channel line driver compatible with supply voltages from 3 V to 5.5 V. Since the MAX3232E does not provide isolation, the Broadcom ACFL-6211T-000E optocoupler [33] was added between the TTL signaling side of the serial transceiver and the microcontroller. Notably, a 3.3 to 5 V signal must be sent through the serial cables and the DB9 connector to power the MAX3232 and its isolated side of the optocoupler.

Lastly, there are the clock, programming, debugging, and reset subsystems of the microcontroller. Fig. 2 illustrates the 8 MHz crystal used for the High-Speed External (HSE) clock, which is multiplied to 72 MHz as the microcontroller’s internal system clock. An optional 32.768 kHz crystal footprint is available for the Low-Speed External (LSE) oscillator, supporting future real-time clock-based functionalities. The main DAQ board provides support for programming and debugging the microcontroller using a variety of tools for ARM devices via two connectors. It has a standard 20-pin JTAG header, which is compatible with the Serial Wire Debug (SWD) interface and also supports Serial Wire Output (SWO) basic Trace. Alternatively, a Cortex Debug+ETM mini-header provides full Trace capability, enabling advanced real-time debugging features, including instruction tracing and data watchpoints, which help troubleshoot time-sensitive or complex firmware. Finally, the main DAQ board features a conveniently placed physical push-button connected to the microcontroller’s hardware reset line. Although not intended for routine operation, it allows for a manual reset, which is helpful during development and debugging.

### 2.2 Pressure transducer board and ignition board description

**Fig. 3:**
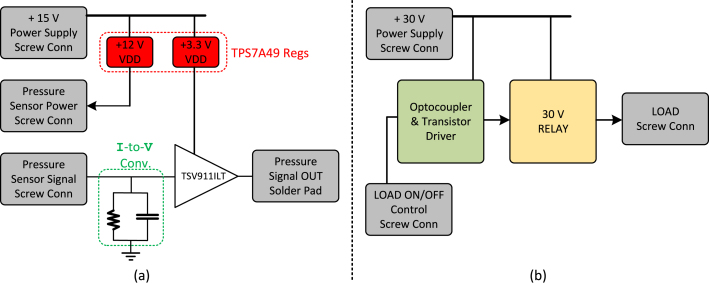
Diagram of (a) the pressure sensor board and (b) the igniter activation board.

Fig. 3 depicts a diagram illustrating the relation between the components and sub-systems of the pressure sensor board and the igniter activation board, respectively, in (a) and (b).

The pressure sensor board includes two TPS7A49DGNT linear voltage regulators, both powered by the same 15 V DC supply that feeds the main DAQ board. The first regulator delivers a stable 12 V output to power the pressure sensor, while the second provides a 3.3 V supply for the remaining circuitry on the board.

The sensor’s 4–20 mA current output is converted into a voltage signal using a passive current-to-voltage (I-to-V) conversion circuit, as shown in Fig. 3(a). This signal is then buffered by a TSV911LT operational amplifier, which also serves to clamp the voltage slightly below 3.3 V, thereby protecting the ADC input and microcontroller frontend on the main DAQ board from over-voltage conditions.

The igniter activation board, shown in Fig. 3(b), is powered by a dedicated 30 V DC power supply. An SPDT relay triggers the igniter upon receiving an activation signal from the isolation stage, which replicates the control signal generated by the microcontroller’s GPIO port. The combination of an independent power source and optical isolation improves operational safety by electrically decoupling the ignition circuitry from the control system, thereby preventing inadvertent ignition due to noise-induced transients.

Detailed schematics and comprehensive hardware assembly instructions are provided in Section 5.

### 2.3 Broader applicability of the system

Although originally developed for static testing of small solid-propellant rocket engines, the DAQ system’s modular design, robust signal conditioning, and cost-effective implementation make it highly adaptable to a variety of experimental and industrial contexts. This flexibility extends its utility beyond the academic aerospace community into several other domains of research and engineering. The following points highlight potential applications:

• **Support for amateur rocketry and educational teams:** Globally, numerous university teams and amateur rocketry groups engage in motor and propellant design. This DAQ system offers an affordable, robust, and safe platform to support static firings, aiding in the validation of thrust curves, combustion efficiency, and burn time in controlled environments.
• **Facilitating propulsion R&D in small aerospace startups:** Startups focused on propulsion innovation, such as the Brazilian company *Edge of Space*, can benefit from a cost-effective and scalable system for rapid prototyping and validation of commercial rocket motor designs.
• **Versatile analog front-end for cross-disciplinary laboratory testing:** The inclusion of a programmable gain amplifier (PGA308) and 16-bit Sigma-Delta ADCs supports high-resolution measurements and broad sensor compatibility, making the hardware suitable for diverse experimental needs.
• **Ideal platform for hands-on engineering education and instrumentation training:** The open design, use of commercial off-the-shelf components, and integration with tools like LabVIEW make it an excellent teaching resource.
• **Strain-gauge-based and pressure transducer applications:** The system’s compatibility with strain-gauge-based sensors and pressure transducers enables easy adaptation to various applications, such as structural and geotechnical monitoring in civil engineering [27], [29]. These include monitoring structural loads, bridge stresses, and soil pressure in geotechnical studies, as well as lysimeter and long-term environmental monitoring.

## 3 Design files summary

All files needed to replicate and implement the data acquisition system designed in this work are available on Mendeley Data in an open-source file format, under the Creative Commons Attribution-NonCommercial 4.0 (CC BY-NC 4.0) license. Academic derivative works are permitted under this license, including adaptations such as modified components, integration of new sensors, or migration to alternative software frameworks, provided proper credit is given and the use remains non-commercial.

The hardware files were developed using Altium Designer Professional version 23.5.1, under an educational license. Each .zip file corresponding to the respective Printed Circuit Board (PCB) contains the schematics, PCB and Schematic libraries, PCB project, and all generated files used to manufacture the boards, following the design constraints for JLCPCB (the chosen PCB manufacturer). In addition, a detailed bill of materials (BOM) for each PCB is also available, containing the specifications of all components used.

The software files for the project were developed using the STM32 Development Tools, including the Integrated Development Environment for STM32 (STM32CubeIDE) and STM32Cube initialization code generator (STM32CubeMX), in version 6.12.1. The project utilized the STM32CubeF3 firmware package version V1.11.0, and the code was compiled using the GNU Tools for STM32 toolchain (version 12.3.rel1). The code was written using the HAL library and based on the documentation of the components used. In order to be user-friendly, the code includes brief comments regarding its main functionalities, already validated in field tests. For the user interface, the Virtual Instrument files developed without commercial purpose using LabVIEW, under the Community Edition 2022 Q3, for controlling the DAQ from a computer via serial communication, are included.

At last, experimental data collected through the data acquisition system built, including noise characterization and bench tests of a small rocket engine, is also available. For possible comparisons, data collected with the commercial system replaced by this hardware was also made available. Photos and videos of the test bench used to carry out the tests are also available.

**Table:** 

Design filename	File type	Open source license	Location of the file
*VECTOR_II_DAQ_MAIN_BOARD.zip*	*Altium Designer Project*	*CC BY NC 4.0*	https://doi.org/10.17632/c2w7759sd3.2.

*VECTOR_II_DAQ_PRESSURE.zip*	*Altium Designer Project*	*CC BY NC 4.0*	https://doi.org/10.17632/c2w7759sd3.2.

*VECTOR_II_DAQ_TRIGGER.zip*	*Altium Designer Project*	*CC BY NC 4.0*	https://doi.org/10.17632/c2w7759sd3.2.

*VECTOR_II_DAQ_BOMs.zip*	*BOM Spreadsheet*	*CC BY NC 4.0*	https://doi.org/10.17632/c2w7759sd3.2.

*VECTOR_II_DAQ_SOFTWARE.zip*	*STM32 Project*	*CC BY NC 4.0*	https://doi.org/10.17632/c2w7759sd3.2.

*VECTOR_II_DAQ_LabVIEW.zip*	*LabVIEW Virtual Instrument*	*CC BY NC 4.0*	https://doi.org/10.17632/c2w7759sd3.2.

*VECTOR_II_DAQ_ACQUIRED_SAMPLE_DATA.zip*	*Sample Data*	*CC BY NC 4.0*	https://doi.org/10.17632/c2w7759sd3.2.

*VECTOR_II_DAQ_MEDIA.zip*	*Test Bench Media*	*CC BY NC 4.0*	https://doi.org/10.17632/c2w7759sd3.2.

VECTOR_II_DAQ_MAIN_BOARD.zip - Altium Designer Project for the main DAQ board.

VECTOR_II_DAQ_PRESSURE.zip - Altium Designer Project for the pressure transducer conditioning PCB.

VECTOR_II_DAQ_TRIGGER.zip - Altium Designer Project for the igniter trigger PCB.

VECTOR_II_DAQ_BOMs.zip - Detailed Bills of Materials for each PCB manufactured.

VECTOR_II_DAQ_SOFTWARE.zip - STM32CubeIDE project written in C for the developed system.

VECTOR_II_DAQ_LabVIEW.zip - LabVIEW virtual instrument files for the Graphical User Interface develop to control the DAQ.

VECTOR_II_DAQ_SAMPLE_DATA.zip - Sample data collected under same conditions using the developed and previous commercial DAQ for comparison purposes.

VECTOR_II_DAQ_MEDIA.zip - Compilation of photos and videos of the Vector II Project test bench.

## 4 Bill of materials summary

The bill of material was divided into sublists, referring to each of the subsections of the data acquisition system. Tables 1, 2, and 3 list the partial BOMs for replicating the main data acquisition, the pressure sensor conditioning, and the igniter trigger hardware, respectively. Details about all resistors and capacitors used are available for access on Mendeley Data: https://doi.org/10.17632/c2w7759sd3.2. The components are general-purpose electronics that can be sourced from various local and international suppliers.

**Table 1:** Data acquisition system hardware bill of materials.

Designator	Component	Number	Cost per unit	Total cost	Source	Material type
U1, U2, U4, U6	TPS7A4901DGNT	4	$3.28 USD	$13.12 USD	https://www.digikey.com/en/products/detail/texas-instruments/TPS7A4901DGNT/2380231	Semiconductor

U3	STM32F373VCT6	1	$9.18 USD	$9.18 USD	https://www.digikey.com/en/products/detail/stmicroelectronics/STM32F373VCT6/3598128	Semiconductor

U5	MAX3232EIDR	1	$1.29 USD	$1.29 USD	https://www.digikey.com/en/products/detail/texas-instruments/MAX3232EIDR/1089416?s=N4IgTCBcDaILIEEAaBmMaCiBJAIgJRAF0BfIA	Semiconductor

U7	PGA308AIDGSR	1	$4.44 USD	$4.44 USD	https://www.digikey.com/en/products/detail/texas-instruments/PGA308AIDGSR/2047524	Semiconductor

U8	REF3430IDBVR	1	$2.49 USD	$2.49 USD	https://www.digikey.com/en/products/detail/texas-instruments/REF3430IDBVR/8037904?s=N4IgTCBcDaIEoFEBiBmALCgDASQCICEA1OEAXQF8g	Semiconductor

U9	REF3450IDBVR	1	$2.49 USD	$2.49 USD	https://www.digikey.com/en/products/detail/texas-instruments/REF3450IDBVR/8635317?s=N4IgTCBcDaIEoFEBiBmALAVgAwEkAiAQgGpwgC6AvkA	Semiconductor

U10	ACFL-6211T-000E	1	$7.57 USD	$7.57 USD	https://www.digikey.com/en/products/detail/broadcom-limited/ACFL-6211T-000E/5824984?s=N4IgTCBcDaIIIGEBiAZAtANjARmwFTQAZiBREAXQF8g	Semiconductor

X1	ATS080BSM-1	1	$0.55 USD	$0.55 USD	https://www.digikey.com/en/products/detail/cts-frequency-controls/ATS080BSM-1/2292836	Other

X2	AB26T-32.768KHZ	1	$0.29 USD	$0.29 USD	https://www.digikey.com/en/products/detail/abracon-llc/AB26T-32-768KHZ/675227	Other

J1, J2, J3	2x1 Screw Conn	3	$0.39 USD	$1.17 USD	https://www.digikey.com/en/products/detail/phoenix-contact/1935161/568614	Other

J4	FTSH-110-01-L-D-K	1	$3.51 USD	$3.51 USD	https://www.digikey.com/en/products/detail/samtec-inc/FTSH-110-01-L-D-K/6678192	Other

J5	DB9 Female Conn	1	$0.83 USD	$0.83 USD	https://www.digikey.com/en/products/detail/assmann-wsw-components/A-DF-09-A-KG-T2S/1241800	Other

J6	10x2 Male Header	1	$1.03 USD	$1.03 USD	https://www.digikey.com/en/products/detail/cnc-tech/3220-20-0100-00/3883663?s=N4IgTCBcDaIMxjABgLTJUgjE1OQF0BfIA	Other

B1	SPST Switch	1	$0.37 USD	$0.37 USD	https://www.digikey.com/en/products/detail/apem-inc/ADTSM644NVTR/1798476?s=N4IgTCBcDaIIIBEAqBlAsgNgCxYHIDUkAlEAXQF8g	Other

D1, D2	BAT54	2	$0.14 USD	$0.28 USD	https://www.digikey.com/en/products/detail/diotec-semiconductor/BAT54/13163463	Semiconductor

R1 …R32	Various SMD resistors	32	$0.1 USD	$3.2 USD	https://www.digikey.com/en/products/filter/chip-resistor-surface-mount/52	Ceramic

C1 …C55	Various SMD ceramic capacitors	55	$0.1 USD	$5.5 USD	https://www.digikey.com/en/products/filter/ceramic-capacitors/60	Ceramic

**Table 2:** Pressure Sensor hardware bill of materials.

Designator	Component	Number	Cost per unit	Total cost	Source	Material type
U1, U2	TPS7A4901DGNT	2	$3.28 USD	$6.56 USD	https://www.digikey.com/en/products/detail/texas-instruments/TPS7A4901DGNT/2380231	Semiconductor

U3	TSV911ILT	1	$0.92 USD	$0.92 USD	https://www.digikey.com/en/products/detail/stmicroelectronics/TSV911ILT/1578404	Semiconductor

J1, J2	2x1 Screw Conn	2	$0.39 USD	$0.78 USD	https://www.digikey.com/en/products/detail/phoenix-contact/1935161/568614	Other

R1 …R5	Various SMD resistors	5	$0.1 USD	$0.5 USD	https://www.digikey.com/en/products/filter/chip-resistor-surface-mount/52	Ceramic

C1 …C12	Various SMD ceramic capacitors	12	$0.1 USD	$1.2 USD	https://www.digikey.com/en/products/filter/ceramic-capacitors/60	Ceramic

**Table 3:** Igniter trigger hardware bill of materials.

Designator	Component	Number	Cost per unit	Total cost	Source	Material type
U1, U2	EL817	2	$0.38 USD	$0.76 USD	https://www.digikey.com/en/products/detail/everlight-electronics-co-ltd/EL817/2693260	Semiconductor

RY1, RY2	G5LE-14-DC24	2	$1.56 USD	$3.12 USD	https://www.digikey.com/en/products/detail/omron-electronics-inc-emc-div/G5LE-14-DC24/280370	Other

Q1, Q2	BC547ATA	2	$0.24 USD	$0.48 USD	https://www.digikey.com/en/products/detail/rochester-electronics-llc/BC547ATA/13466514	Semiconductor

J1 …J5	2x1 Screw Conn	5	$0.81 USD	$4.05 USD	https://www.digikey.com/en/products/detail/phoenix-contact/1935776/2513905?s=N4IgTCBcDaIIwE4DMBWA7GgbCAugXyA	Other

D1, D2	1N4007GP	2	$0.13 USD	$0.26 USD	https://www.digikey.com/en/products/detail/diotec-semiconductor/1N4007GP/22192746	Semiconductor

R1 …R4	Various TH resistors	4	$0.1 USD	$0.4 USD	https://www.digikey.com/en/products/detail/yageo/CFR-25JB-52-10K/338?s=N4IgTCBcDaIMIDEBKBaMBWAUgIResKAjAAwDSIAugL5A	Ceramic

## 5 Build instructions

**Fig. 4:**
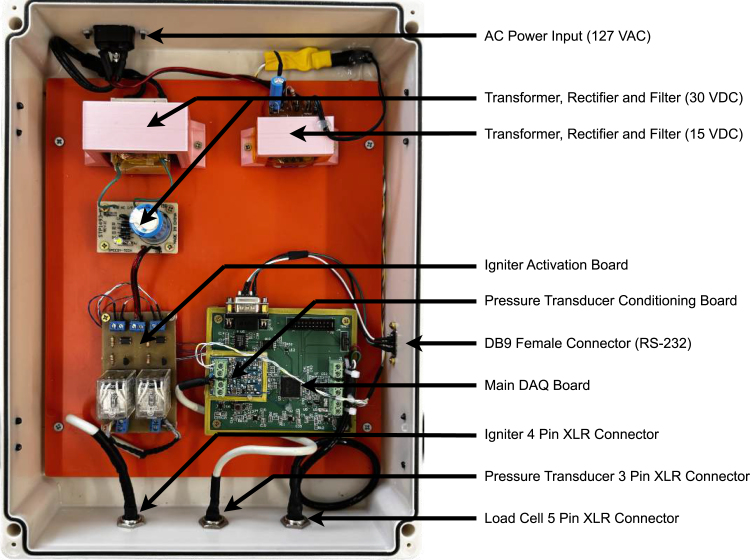
Close-up view of the hardware assembly.

Fig. 4 depicts the entire data acquisition system and its auxiliary parts. It can be broken down into five distinct parts and the input/output connectors that interface with those parts:

1. The main DAQ Module, which has the load cell instrumentation, the serial interface, and its power supply circuit;
2. The Pressure Transducer Conditioning Module and its Power Supply Circuit;
3. The power supply of the Main DAQ Module and the Pressure Transducer Conditioning;
4. The Igniter Activation Module;
5. The power supply of the Igniter Module.

It is essential to emphasize some design decisions before specifying the instructions for each part. Technically, although some parts of the system could be integrated into a single printed circuit board, this would have reduced the prototype’s scalability, potentially leading to increased costs if a sensor or specific hardware had to be changed. Consequently, we chose to keep the pressure sensor conditioning electronics separate from the main DAQ and the igniter module as a separate circuit. Nonetheless, from a technical perspective, one could integrate the multiple parts of the design into a single electronic circuit.

Regarding the system’s physical structure, a commercial external power distribution box was adapted to accommodate all system elements. All cuts, holes, and threads were manually made in the enclosure’s sides to hold the panel connectors. From the user’s point of view, connectors for power supply, load cell, pressure transducer, igniters, and serial interface are easily accessible. It is worth noting that each connector has a unique number of wires and plug patterns, ensuring that the user cannot incorrectly connect them.

The AC power input is a typical power line connector with a direct electrical connection to the transformers. Two transformers and their capacitor-filtered full-wave rectifiers provide isolated 15 V and 30 V DC power, respectively. Optionally, two independent battery packs can also provide DC power to the system. Powering from a battery would involve reconnecting the PCBs’ power supply screw terminal blocks from the transformer circuits to the batteries, removing the need of an AC power line in field tests.

The serial interface features a DB9 receptacle cable connector mounted on the lateral right side panel of the box, with its counterpart connected to the main DAQ board. The load cell signal, pressure sensor signal, and up to two igniter activation signals are accessible via screw terminal blocks on their respective PCBs. These signals are then routed through cables to their corresponding XLR connectors, which are mounted on the bottom side panel of the enclosure. The connector configuration consists of a 5-pin XLR for the load cell, a 3-pin XLR for the pressure sensor, and a 4-pin XLR for the igniters. The microcontroller debug connectors are the only interfaces not accessible through the enclosure panels, requiring, intentionally, direct access on the PCB, as they are not intended for use during actual engine testing.

The remainder of this section is organized into step-by-step instructions detailing each part of the electronic design. To facilitate understanding of the design, each section depicts a particular part of the electronics rather than each printed circuit board.

### 5.1 Main DAQ board — microcontroller system instructions

As mentioned in the Hardware description section, the STM32F373VC MCU was primarily chosen due to its integrated 16-bit Sigma-Delta A/D converter (SDADC) and a free and user-friendly software development interface provided by STMicroelectronics. The Sigma-Delta architecture offers excellent resolution and linearity, complying with the system’s sampling rate of 1000 samples per second. This acquisition rate was defined based on prior experience from the Vector Project [10], and it aligns with the typical bandwidth of resistive load cells, which usually do not exceed a few hundred hertz [34]. Therefore, the analog filter has a cutoff frequency of 100 Hz, and a sampling frequency of 1000 Hz is used in the SDADC. The latter is equivalent to five times the minimum value to meet the Nyquist criterion.

Another notable feature of this peripheral is the inclusion of a gain amplifier integrated with the microcontroller’s SDADC. This amplifier supports analog gain settings of 0.5, 1, 2, 4, and 8, as well as digital gain factors of 16 and 32. When combined with the three programmable amplification stages of the PGA308, this constitutes a fourth stage of gain applied to the load cell signal, providing enhanced flexibility for adapting to a wide range of sensor outputs and measurement conditions. The converter also has multiple operating modes: Differential, single-ended offset, and Single-ended zero-reference. Based on the DAQ design, the zero-reference mode is used since the load cell and the pressure sensor do not perform negative measurements. Nonetheless, having such configurations available is important for modularity and possible future upgrades or sensor and hardware changes.

The microcontroller is clocked by an external 8 MHz crystal, which is connected to its High-Speed External (HSE) oscillator, as shown in Fig. 2. This external clock signal is internally multiplied by a Phase Locked Loop (PLL) stage to 72 MHz, which is then used as the microcontroller’s main system clock (SYSCLK). The main DAQ board features a footprint for the optional soldering of a 32.768 kHz crystal, which is intended to drive the microcontroller’s Low-Speed External (LSE) oscillator. This enables the use of the real-time clock (RTC) peripheral, which may add future functionalities dependent on accurate timekeeping. The crystals have associated external capacitors as part of the Pierce oscillator implementation. Their values are given directly by the manufacturer’s datasheet, and the current-limiting resistor value can be calculated using [35].

**Fig. 5:**
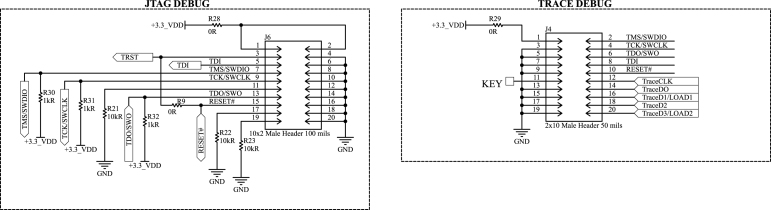
Debug connectors schematic.

Furthermore, the wide availability of programming and debugging tools for ARM devices allowed the implementation of several debugging modes for the microcontroller through two connectors soldered into the printed circuit board, as illustrated in Fig. 5. The first is a standard JTAG interface, accessible via a 20-pin, 2.54mm pitch, dual-row header. This is the most accessible and commercially available method. In addition to JTAG, this connector also supports the Serial Wire Debug (SWD) interface, which is a widely used two-pin protocol for programming and debugging ARM-based microcontrollers, as well as Serial Wire Output (SWO) for basic Trace functionality. For full Trace support, including advanced debugging features such as real-time data streaming and instruction-level execution tracing, a separate 20-pin, 1.27mm pitch dual-row Trace connector is provided. As there are different programming tools available, the design has some 0 Ω resistors, allowing the user to power the microcontroller via the programming tool if required. However, typically, the programming tool and the DAQ board are powered independently of each other. Furthermore, the documentation is unclear about TRST and RESET# pins, and their connection depends on whether one is using the JTAG or Trace debug options; however, R9 is typically not required. It is also worth noting that the pull-up resistors R30, R31, and R32 are required depending on the programming tool.

### 5.2 Main DAQ board — load cell conditioning and signal amplifying

The load cell conditioning circuit has many integrated circuit options. Among those, the PGA308, an auto-zero sensor amplifier with programmable gain and offset, stood out as a suitable solution for the tasks. Furthermore, it has two essential features: a three-stage voltage amplifier and an over-voltage clamp protection. The first is important so that it can provide a fine-tuning of the output voltage for the differential input voltages of the load cell resistive bridge. Besides, the final amplification stage can also be achieved through the use of external resistors, thereby contributing to the minimization of noise in the amplification process. Consequently, given the versatility of the component configurations, it is possible to power the load cell with voltages exceeding the standard limit of 5 V. In this sense, with the larger power supply, it is possible to reduce the total gain required to condition the differential signal, reducing the total acquisition noise. On the other hand, operating the system outside the standard range prevents automatic detection of certain faults, such as input over-scale, placing the responsibility on the user to be aware of this limitation. The over-voltage clamp protection is essential to provide an extra safety feature for the hardware in case of catastrophic failure or explosion during a test that damages the load cell and could lead to an overvoltage in the PGA308 output, protecting the remainder of the DAQ circuit and the microcontroller ADC.

**Fig. 6:**
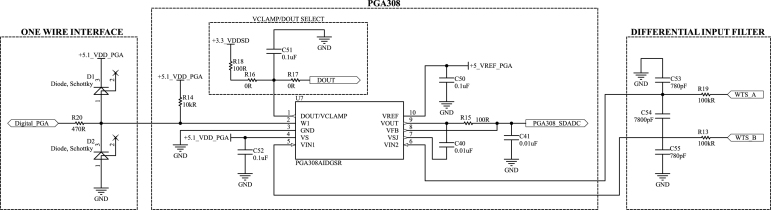
PGA308 circuit schematic.

The load cell conditioning circuit, based on the PGA308, was designed following guidelines and reference designs provided by Texas Instruments, as detailed in the PGA308 User’s Guide [36]. Fig. 6 presents the main section of the schematic. The TPS7A49 regulators, which generate 3.3 V and 5.1 V, are omitted from this schematic since their details are discussed in Section 5.4.

It is worth highlighting the low-pass RC differential filter implemented in the sensor inputs, whose transfer function is represented by

(1) $$\frac{V_{\mathrm{tune}}^{\mathrm{diff}}\left(s\right)}{V_{\mathrm{in}}^{\mathrm{diff}}\left(s\right)}=\frac{1}{1+21sRC},$$

and cutout frequency:

(2) $$f_{\mathrm{cut}}=\frac{1}{42\pi RC},$$

where $V_{\mathrm{in}}^{\mathrm{diff}}$ and $V_{\mathrm{tune}}^{\mathrm{diff}}$ are, respectively, the differential input voltage and the differential voltage after filtering (in volts), R is the resistance of resistors $R_{13}$ and $R_{19}$ (in ohms), C is the capacitance of capacitors $C_{53}$, $C_{55}$, and 10% of the capacitance of $C_{54}$ (in farads), and $f_{\mathrm{cut}}$ is the cutoff frequency of the filter (in hertz).

Using (2) for a cutoff frequency as close as possible to 100 Hz, and defining resistors of 100 kΩ, the resulting commercial capacitors are 780 pF and 7800 pF. These components yield a cutoff frequency of 97.2 Hz.

The Schottky diodes in Fig. 6 are recommended as protection and filter of the serial communication with the microcontroller. In addition, there are two 0 Ω (R16 and R17) resistors to select between two functions: VCLAMP, to limit the amplified output voltage (main use), and DOUT, for future calibration of sensor modules to be added to the printed circuit board. Lastly, we emphasize that the solution for conditioning the load cell, which is powered by an independent adjustable voltage regulator, allows versatility in controlling the power supply from adjustable voltage and with high current capabilities.

Other alternatives are widely available for load cell conditioning circuits, such as the Analog Devices MAX1452 and MAX1454 signal conditioners. These feature an internal temperature compensation circuit, which can benefit applications that require minimizing variations in load cell measurements due to temperature changes. While beneficial for long-duration measurements, this feature is not essential for short rocket engine tests, which typically last only a few seconds and do not allow the temperature to impact the sensor output. Additionally, these ICs have an internal bridge power supply limited to 2.5 mA, which restricts the excitation voltage when driving low-resistance load cells. For instance, the Vector II load cell has a 760 Ω resistance, which would be constrained to 1.9 V, limiting the output range of the signal conditioner. Still, for temperature-sensitive applications that utilize sensors within the power supply range, other signal conditioners can be an excellent alternative to the circuit implemented in this work.

### 5.3 Main DAQ board — RS-232 serial interface

As discussed in Section 2.1, the serial interface of the DAQ system with the external equipment requires either galvanic or optical isolation. Primarily due to the availability of the MAX3232 IC, it was decided to maintain this non-isolated RS-232 driver and add optical isolation using an ACFL-6211T-000E optocoupler. Nonetheless, if one seeks to reproduce the hardware, integrated circuits that encompass isolated RS-232 drivers, such as those from the MAX or ADM families of Analog Devices, are viable options using a single IC. Another possibility is to use a non-isolated RS-232 transceiver with a galvanic isolation IC at the TTL level, such as the ADUM family from Analog Devices. All these combinations have a similar cost, and the choice may also have to consider the maximum data rate of each circuit, which, in our case, was not a concern.

The drawback of using the ACFL-6211T-000E optocoupler is that this specific chip has an inverting logic at the isolated level. Thus, two Schmitt-Trigger inverters, specifically the SN74LVC1G14QDCKRQ1, are seen in the schematic in both the receiver and transmitter lines. Despite having extra components in the hardware, physical space was not an issue, and the SN74LVC buffers are inexpensive and readily available.

**Fig. 7:**
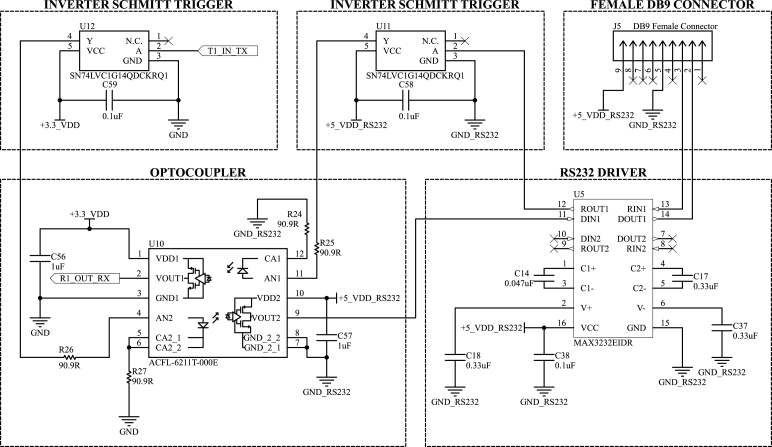
Isolated RS-232 serial interface used in the main DAQ board.

The serial interface schematic is seen in Fig. 7. Due to the isolated interface, the female DB9 connector has a 5 V signal in pin 9, which must come from an external power source. Using an external power source eliminates the need to generate an isolated power supply for the MAX3232, utilizing a typical isolated DC–DC converter, such as the ADUM1500 integrated circuit. Nevertheless, if one seeks to avoid the need for an external power supply in pin 9, that would be the best alternative, albeit at an increased cost.

To avoid introducing unnecessary complications, the chosen RS-232 standard does not use the flow control pins and requires the Tx, Rx, GND, and a 5 V power supply. Additionally, every computer’s power supply has a 5 V available. Nonetheless, if only a 3.3 V is available in the User Interface system, the MAX3232 can also operate at this voltage level, requiring only the adjustment of its capacitors [37].

### 5.4 Power supply system — main DAQ board and pressure transducer board

As described before, the DAQ and the pressure system must have a DC voltage between 12 and 36 V. In the prototype built, this voltage is generated by a 15 V power supply, which includes a transformer, rectifier, and filter. Afterward, all regulators within the electronic design are the TPS7A49 linear circuit from Texas Instruments.

**Fig. 8:**
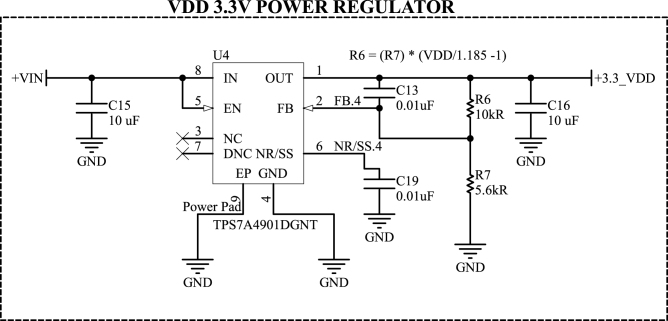
Circuit schematic used in the TPS7A49 linear regulator.

The TPS7A49 is a 36 V, 150 mA, ultralow-noise, adjustable positive linear regulator [32]. Its typical circuit, used in the design, is shown in Fig. 8. The output voltage is adjusted by two external resistors, following the relationship:

(3) $$R_{6}=R_{7}\left(\frac{V_{\mathrm{OUT}}}{V_{\mathrm{FB}}}-1\right),$$

where $R_{6}$ and $R_{7}$ are the feedback resistors that set the output voltage (in ohms), $V_{\mathrm{OUT}}$ is the desired output voltage (in volts), and $V_{\mathrm{FB}}$ is the feedback voltage of 1.185 V.

In addition, the resistance $R_{7}$ must meet a minimum current, given by

(4) $$\frac{V_{\mathrm{FB}}}{R_{7}}\geq 5\;\mu A$$

resulting in a maximum value for resistor $R_{7}$ of 237 kΩ.

Furthermore, the TPS7A49 has a power pad to dissipate heat generated by the dropout voltage, i.e., a terminal under its encapsulation that, in the PCB, must be connected directly to the ground plane through vias. The PCB design also features an upper ground plane shared by all regulators, aiming to improve heat dissipation further. Considering all the specificities for using the component, the manufacturer provides a plan to be followed when routing the printed circuit board.

**Fig. 9:**
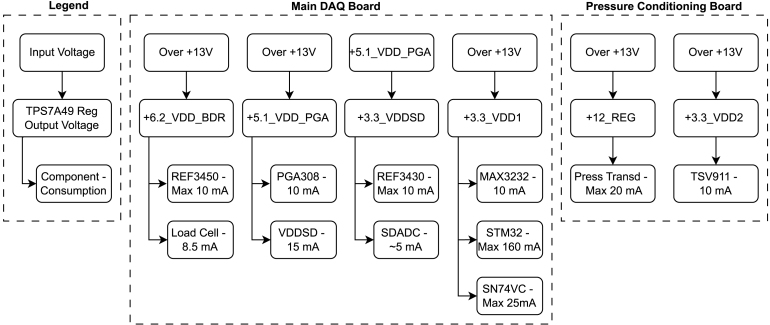
Power diagram and theoretical typical and maximum current consumption of the system’s key components.

The electronic system requires four distinct voltage levels: 12 V, 6.2 V, 5.1 V, and 3.3 V, which are supplied by distinct TPS7A49 linear regulators. The main DAQ board includes four of these regulators, while the pressure transducer board incorporates two. The roles of these voltage levels within the system are explained in Sections 2.1, 2.2, respectively. Fig. 9 summarizes the use of the regulators in the project, which also depicts the theoretical current the system’s key devices could consume.

In addition to the voltage regulators, the system includes two voltage reference integrated circuits: the REF3430 and the REF3450, which provide precise 3.00 V and 5.00 V reference voltages, respectively. The REF3430 is used to supply a stable reference voltage to the microcontroller’s Sigma-Delta ADC, improving measurement accuracy. The REF3450 serves as the voltage reference for the internal analog circuitry of the PGA308 IC.

The reader must note that the design lacks reverse polarity protection diodes or a more sophisticated protection circuit in the input connectors. This potential safety hazard requires attention when assembling all wiring and connections. Future versions of the hardware could have additional protections.

### 5.5 Pressure transducer board — pressure data acquisition

As briefly described in previous sections, secondary hardware acquires the engine’s internal pressure during tests. The pressure transducer in the data acquisition system features a standard current output signal of 4 mA to 20 mA, typically used in industrial control systems. Therefore, the signals from the transducer are first converted to voltage using a simple resistor calculated for the system’s conversion range; in this case, the full scale is 3 V, compatible with the Sigma-Delta ADC input range. Then, an operational amplifier, the TSV911ILT, configured as a buffer, isolates and protects the DAQ. The amplifier isolates the resistor for current–voltage conversion and its respective capacitor for low-pass filtering (anti-aliasing), providing a signal with very low impedance to the microcontroller’s ADC. Furthermore, the converter’s input voltage is limited to the amplifier’s rail-to-rail power supply, ranging from 0 V to 3.3 V, within the ADC’s input limit of 4 V. The TSV911 also features internal input diodes to protect against overvoltage due to the pressure sensor output signal.

**Fig. 10:**
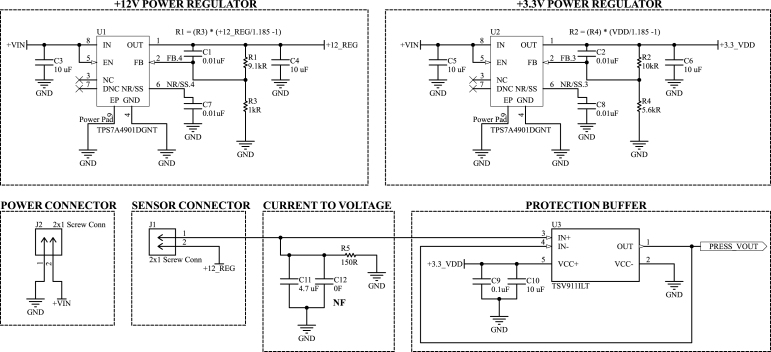
Pressure data acquisition circuit schematic.

The schematic diagram in Fig. 10 shows the complete circuit design. It uses two TPS7A49 voltage regulators to generate 12 V and 3.3 V, respectively, to power the external pressure sensor and the pressure transducer board OpAmp buffer. Furthermore, the resistor used in the current-to-voltage conversion circuit is determined based on the upper voltage limit of the main DAQ board’s analog-to-digital converter input (3 V) and is calculated using Ohm’s law. As a result, a 150 Ω resistor is used.

### 5.6 Main DAQ and pressure transducer printed circuit boards

**Fig. 11:**
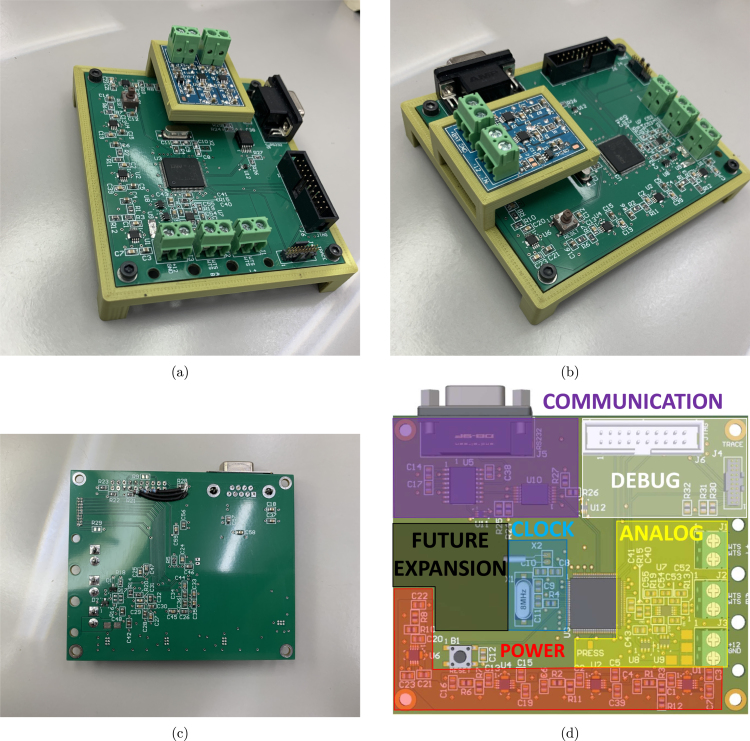
Printed circuit boards result. (a) Top View. (b) Pressure acquisition. (c) Bottom view. (d) Board subsystems defined areas.

A four-layer PCB was developed with all the selected components and schematic diagrams in the main DAQ hardware. It handles all the hardware components while making it intuitive for the final user operation, with well-defined areas per functionality, depicted in Fig. 11(d). The PCB was fabricated by a commercial manufacturer, and its assembly was performed manually in a university laboratory without automation.

Regarding the crucial area of the board for the load cell conditioning and its signal acquisition, the right end side was reserved for the complete PGA308 circuit and the screw terminal block connectors for the sensor. This solution ensures a direct line to the input of the microcontroller’s Sigma-Delta converter, as recommended by Texas Instruments. As evident from the visual representation in Fig. 11, the STM32F373VCT6 microcontroller was positioned at the center of the hardware, facilitating routing to the various regions and components. The large area to the left of the printed circuit board and the various unused microcontroller pins were reserved for later expansions in the data acquisition system, including the pressure sensor.

Due to the high sensitivity of the data acquisition signals, guard planes were implemented on the board, connecting the GND plane to specific nets indirectly. In other words, the planes are not directly connected to the components or their tracks; they only surround them and are subsequently connected to the GND through a small copper mesh. This helps to reduce interference and isolate the circuits.

The secondary PCB, which holds the pressure transducer signal conditioning electronics, is much simpler, and a double-layer PCB was sufficient. This additional board was positioned over the main board using a 3D-printed structure, with the connection to the microcontroller’s SDADC input pin made through a wire routed to a solder pad connection.

Fig. 11 displays images of the final prototype hardware, and (a) and (b) depict both boards. Once again, it is essential to emphasize that this solution, featuring two different boards, ensures safety and modularity. For instance, the pressure transducer PCB could be configured for various pressure ranges or redesigned to suit other transducers without requiring changes in the main DAQ board. However, future versions or definitive hardware for specific sensors might be manufactured and assembled in a single PCB.

### 5.7 Igniters activation module description

**Fig. 12:**
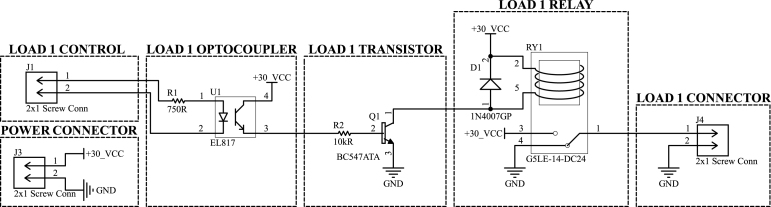
Ignition board circuit schematic.

Fig. 12 shows the schematic of the electronic circuit used to activate the engine igniter charges. The prototype PCB features two identical channels; however, only one is depicted in the schematic for clarity. In the current implementation, the two modules can be used either redundantly or to trigger two independent loads, depending on the propellant architecture adopted [10].

Each subcircuit includes an EL817 optocoupler, which electrically isolates the microcontroller’s I/O pins from the load-driving circuitry. The igniters are activated by a G5LE-14-DC24 relay powered by an independent source of approximately 30 V direct current. A BC547ATA transistor polarized by the optocoupler energizes the relay’s coil, and a flyback diode allows its coil to discharge during turnoff.

This circuit is simple, and many distinct components are readily available and could be used in its design. The most crucial point of the igniter circuit is to have a completely independent power source for extra safety reasons. One must note that turning the relay on at an unwanted moment could trigger the ignition.

Since the relay’s NC (Normally Closed) terminal and one of the igniter’s terminals are permanently connected to the ground, both igniter terminals remain short-circuited to the ground when the relay is de-energized, ensuring a safe default state. The activation signal is routed to an optocoupler, which in turn drives a switching bipolar junction transistor (BJT) that either saturates or cuts off, controlling the current through the relay coil. This configuration ensures that the igniter remains in a fail-safe state by default and with galvanic isolation between the control logic and the high-current ignition circuit, significantly reducing the risk of accidental ignition due to electrical faults or noise. Nonetheless, the igniter cable should only be connected to the system once all preparations and additional safety measures are in place to proceed with the engine test.

### 5.8 LabVIEW virtual instrument

**Fig. 13:**
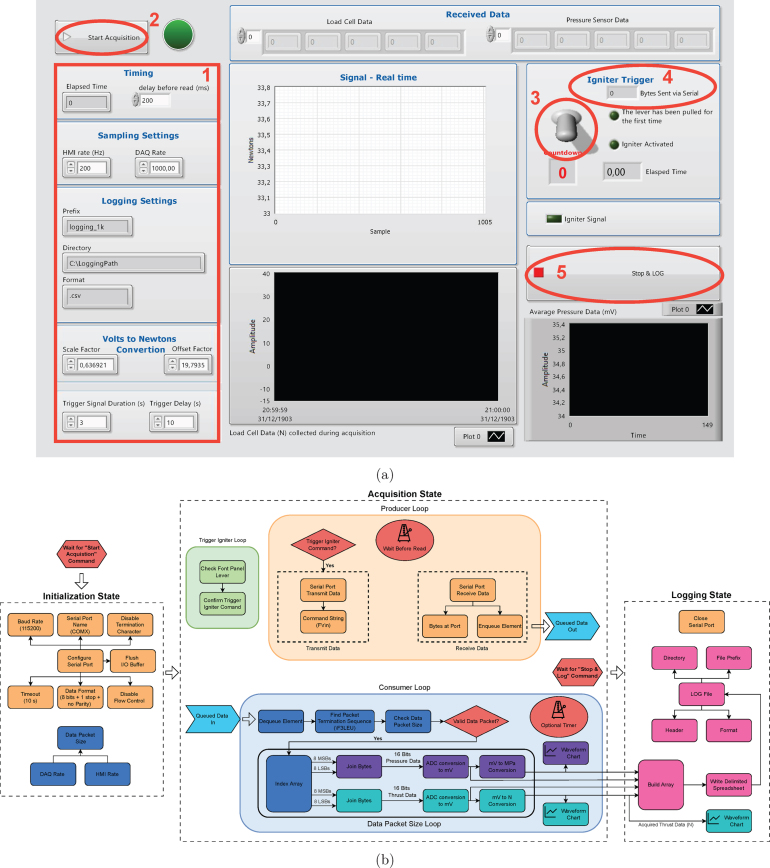
LabVIEW Virtual Instrument. (a) Front panel GUI. (b) Block diagram of execution logic.

A graphical user interface (GUI) was developed in LabVIEW to control and monitor the data acquisition process. From the user’s perspective, the most critical element is the front panel (Fig. 13(a)), which displays real-time plots of thrust and pressure acquired from the DAQ system. In addition to viewing the acquired signals, the user can control igniter activation and configure parameters such as logging options and acquisition timing. However, the latter must be adjusted along with the embedded software since LabVIEW currently does not support dynamically changing the system’s acquisition and transmission rate.

Fig. 13(b) illustrates the block diagram of the developed Virtual Instrument (VI), structured into three sequential execution states using the *Flat Sequence* structure. Upon launch, the system remains idle until the user activates data acquisition by pressing the “Start Acquisition” button, indicated by 2 in Fig. 13(a). Once triggered, the VI initializes the serial communication port, as indicated by the orange blocks in Fig. 13, with parameters aligned with those defined in the embedded software: 8 data bits, 1 stop bit, no flow control, and a baud rate of 115200. This configuration supports the transmission of 240-bit packets at 200 Hz, equivalent to 48000 bits/s, well within the capabilities of the transmission hardware and providing a suitable safety margin.

To enhance robustness, a 10-s timeout is configured to tolerate brief communication interruptions. Termination character detection is disabled since a custom multi-character sequence is employed to mark the end of each data packet, minimizing the risk of misinterpreting valid data as a terminator. Once the interface is configured, the host computer’s I/O buffer is flushed to discard any residual data before acquisition begins.

The second execution state corresponds to the acquisition process, structured into three independent *while* loops: the producer loop, the consumer loop, and the trigger igniter loop. The producer–consumer structure is fundamental for achieving reliable real-time data acquisition in LabVIEW. The producer and consumer loops are interconnected via a “First In, First Out” (FIFO) queue, ensuring that data packets are transferred without delays or loss. The producer loop reads all available data from the host computer’s serial port at user-defined intervals on the front panel and enqueues the data. Concurrently, the consumer loop continuously retrieves and processes data from the queue.

The “wait before read” interval, which determines the timing of data reads from the serial port, must be selected based on the host computer’s capabilities. In this system, a 5 ms delay, matching the 200 Hz transmission period, was found to ensure stable performance without overloading the processor, while still maintaining a low-latency visual response for the user.

The producer loop is also responsible for transmitting the ignition command string via the serial interface to the main DAQ hardware. The ignition is initiated by the user through a lever control on the GUI front panel, indicated by circle 3 in Fig. 13(a), and is managed independently by the trigger igniter loop. This loop handles a two-step validation: it monitors the user input and prompts for a secondary confirmation before setting a flag. Once this flag is detected by the producer loop, it is interpreted as authorization to transmit the ignition command to the hardware.

The consumer loop, highlighted in blue in Fig. 13(b), dequeues the received data and uses the *Match Pattern* function to search for the termination sequence, which is configured as *\F3LEU*. The ADC data is transmitted in binary format to reduce packet size and avoid overloading the serial interface. Upon locating the terminator, any subsequent data is reintroduced into the detection logic and concatenated with new incoming FIFO data. The identified data packet is then verified by checking its size before being processed in the internal data packet size loop. Within this loop, the data array is indexed to separate the most significant and least significant bytes for both thrust and pressure readings. These values are combined to reconstruct the 16-bit integers corresponding to the data of analog-to-digital converters.

Next, the raw data is converted to millivolts based on the ADC configuration and then scaled to physical units, megapascals (MPa) for pressure and newtons (N) for thrust, using the previously determined calibration equations. The signals are displayed in *waveform charts* on the front panel for real-time user visualization. This architecture is highly modular: adding new sensors requires only adjusting the packet indexing and replicating the conversion logic for the additional data fields.

It is worth noting that the consumer loop does not strictly require a minimum execution time control. However, a delay of 1 ms is recommended to prevent excessive CPU usage on the host-computer. This delay corresponds to the processing time per data packet, so reducing it decreases the latency experienced by the user, improving real-time responsiveness.

The data acquisition process continues until the user presses the “Stop & LOG” button on the GUI, indicated by the ellipse area 5 in Fig. 13(a). At this point, the producer loop is terminated, while the consumer loop continues processing any remaining data in the queue. The VI then transitions to its final execution state (logging stage), shown on the right side of the diagram in Fig. 13(b), and closes the serial port.

For data logging, the directory, filename prefix, and format defined by the user on the front panel, indicated by the logging settings area 1 in Fig. 13(a), are used to generate a log file, typically in CSV format. All acquired data is saved using LabVIEW’s *Write Delimited Spreadsheet* function. Additionally, a graph showing the complete thrust profile is displayed at the center of the GUI to provide an immediate visual summary of the test results.

In summary, the developed Virtual Instrument integrates user interaction, DAQ control, igniter triggering, and data logging in a modular and adaptable interface. Although the VI was developed using the LabVIEW Community Edition, only fundamental software features were employed, ensuring compatibility with other license types. Full implementation details are available in the design files, but it is worth emphasizing that the architecture can easily accommodate different sensor types or packet structures, provided that the data processing logic is adjusted accordingly.

## 6 Operation instructions

### 6.1 Software development

The current version of the Graphical User Interface does not support embedded software updates. As a result, any customization of the acquisition system requires direct modification of the microcontroller software. To facilitate future development and maintenance, the embedded software was structured around a single main function alongside with interruption routines. This design follows a straightforward approach, with explicit peripheral initialization, hardware configuration, and a central loop that manages continuous data acquisition and communication. The resulting architecture is both modular and accessible, enabling users with varying levels of embedded programming experience to extend or customize functionalities with minimal complexity.

Upon startup, the system performs peripheral initialization, including GPIO, direct memory access (DMA), Sigma-Delta ADCs, timers, and UART interfaces. The PGA308 signal conditioning device is configured by loading predefined register values. Simultaneous conversions of pressure and thrust signals are initiated via two separate Sigma-Delta converters triggered sequentially by a timer, running at a sampling rate of 1000 Hz.

The software’s main loop monitors several flags to coordinate operations:

• **Ignition Command Reception:** A UART reception flag (UART_RX_CPLT_FLAG) signals when a 3-byte command is received from the host. The command buffer is checked for a specific ignition sequence (‘F\r\n’). Upon detection, digital output pins controlling the ignition loads are set high, and a timestamp is recorded to manage ignition duration control via timing.
• **Igniter Deactivation:** A timer-based check disables the ignition loads after a fixed 3-s interval by resetting the corresponding GPIO pins.
• **Sensor Data Acquisition and Transmission:** The software uses an interrupt-driven callback (HAL_SDADC_InjectedConvCpltCallback) to set an user defined flag (SDACD_FLAG) whenever new ADC data are ready. Raw ADC values for thrust and pressure are stored in temporary buffers at 1000 Hz. To avoid overwhelming the host computer and serial interface, data are buffered into groups of five samples and sent as a binary packet at a 200 Hz transmission rate. Each packet includes a fixed termination sequence (0x55454CF3) for synchronization, although additional security measures, such as checksums or parity bits, could be implemented if required. The UART transmission is performed using DMA for efficient, non-blocking data transfer, with completion indicated by a callback (HAL_UART_TxCpltCallback). Preliminary tests confirmed that the host computer running LabVIEW could reliably handle real-time acquisition up to 500 Hz without errors or data packet loss. Nevertheless, the 200 Hz transmission rate was deliberately chosen to ensure robust performance and avoid overloading the host system’s serial input buffer. This decision slightly reduces the update rate of the real-time display but does not introduce perceptible latency to the user.

The UART receive process is handled with interrupt-driven callbacks (HAL_UART_RxCpltCallback), automatically restarting reception after each packet to ensure continuous command monitoring without blocking the main loop.

Although the current implementation is tailored to a fixed configuration of two Sigma-Delta ADCs and specific signal conditioning hardware, the software’s clear and linear structure facilitates adaptation. Additional sensor types can be integrated by replicating existing acquisition and buffering logic with minor changes to the DMA or ADC initialization routines.

The embedded software includes a modular driver written specifically for the PGA308 signal conditioning device, which encapsulates all communication details through a half-duplex UART interface. The driver abstracts register read and write operations, device configuration, and status verification, providing a clean and reusable interface for the main application. It also exemplifies the modular design approach adopted in the software, facilitating maintainability and scalability.

Integrating additional sensors or replacing hardware components — such as higher-resolution ADCs or sensors communicating via I^2^C or SPI — can be achieved by developing similar dedicated drivers that interface with the main data acquisition and transmission logic without extensive software restructuring.

At this stage, no hardware abstraction layer (HAL) beyond the STM32Cube HAL is used, meaning modifications must respect the STM32 peripheral structure. However, because acquisition, transmission, and control routines are encapsulated in distinct and independently testable modules, scalability is feasible without restructuring the entire software.

This software architecture ensures reliable real-time acquisition and transmission of sensor data, alongside controlled ignition activation, balancing performance demands and resource constraints typical of embedded systems used in rocket engine testing.

### 6.2 The PGA308 programmable gain amplifier

The PGA308 has multiple registers to control its features, such as the gain, offset voltage, fault detections, over and under-scale limits, and output clamp protection. Based on the configuration of these registers, different errors can be detected and are reported directly to the alert register, or a combination of faults could trigger a specific alert from the truth table available in the User Guide [36]. Hence, the microcontroller must initialize these settings after the startup or load them directly into the one-time programmable memory.

As a protection mechanism against electrical or configuration-related faults, the PGA308 output can saturate positively or negatively to warn severe errors and faults. Therefore, prior analyses to determine the gains and offsets at each stage are essential for the project’s success. Accounting for that, the document in [36] provides a step-by-step example for defining the parameters. Additionally, it is possible to do this automatically using the development software provided by the manufacturer Texas Instruments [38]. The software consists of a GUI in which it is possible to select all the desired configurations and inform the characteristics of the sensor to be used. As a result, it automatically detects configuration errors and provides the values to be loaded into the PGA308’s registers.

As a future feature of the DAQ system, the GUI in the LabVIEW virtual instrument could allow the user to define the parameters and register values. Then, the instrument would automatically communicate with the microcontroller and set the values in the PGA308’s registers.

### 6.3 System calibration

The sensors and the DAQ system hardware must be calibrated before the experiments to ensure accuracy. The test bench utilizes a wheel spoke (donut) FCMA 200 kg load cell; however, any circular load cell with the same weight rating is suitable for the engine in question. In different systems or depending on the mechanical design of the engine, other load cells might be better suited.

The FCMA sensor has an output of 2 mV/V and an excitation voltage of 6.2 V, which is relevant to configuring the PGA308. First, the offset correction is set without any load, and the PGA308 is programmed to cover the entire 3 V range of the Sigma-Delta ADC. Afterward, starting with no weight, a sequence of known masses is applied one at a time. Once the maximum weight is reached, the masses are removed in reverse order, returning to the initial weight. At each weight level, ADC data is recorded for approximately 15 s and subsequently converted to its corresponding millivolt values.

After calculating the mean value of the data collections for every weight point, it is possible to use a linear regression relating the weight or force applied to the load cell and the corresponding voltage from the ADC, following the relationship

(5) $$F=a_{0}\times V_{\text{ADC}}-b_{0},$$

where $a_{0}$ and $b_{0}$ are the linear calibration terms, $V_{\text{ADC}}$ is the input voltage to the Sigma-Delta ADC (in volts), and F is the resulting force (in newtons).

Alternatively, a machine, such as a hydraulic press, could be used to perform an automated procedure to calibrate the load cell instrumentation. However, that would require an expensive apparatus that is not always easily and readily available. Automated calibration is beyond the scope of this work, which focuses on the development of a low-cost, in-house data acquisition system. The system has demonstrated stable behavior across multiple calibrations, with no significant variation observed, reinforcing manual calibration as a practical and effective solution.

Calibrating the pressure transducer presents a more complex procedure, as acquiring a reference device is costly, whereas constructing a suitable calibration apparatus is not a trivial task. Since no dedicated calibration was performed, the pressure data was bias-corrected using the known atmospheric pressure at the altitude of the test bench location, and the scale factor was determined based on the manufacturer-provided information. Notably, setting the appropriate conversion range for the 4–20 mA signal to its corresponding voltage level, as discussed in a previous section, is essential to maximize measurement accuracy. Moreover, prior work has demonstrated that coherent and reliable results can still be obtained in the absence of formal calibration [11].

### 6.4 Procedure and safety measures for experiments with the test bench

As the main points regarding the initial configuration of the system have been described in detail, the procedure for carrying out an experiment is described using step-by-step instructions, highlighting safety hazards:

1. With the system disconnected from the power supply, connect the sensors to their respective XLR connectors;
2. Before powering up the hardware, check the polarity of the power supply with a DC voltage greater than 12 V since there are no protection diodes;
3. Without connecting the pyrotechnic (igniters) charges, supply the igniter drive system with a DC voltage greater than 30 V. Use a multimeter to verify that the supply voltage of the relay circuit is within the expected range and that the relay output is not shorted;
4. Plug the DB9 connector for the serial interface, providing power for the isolated hardware components and taking into account the pinout provided in Fig. 7;
5. After powering the receiver hardware, plug the serial cable into an RS-232-compatible port on a host computer;
6. Run the virtual instrument using LabVIEW software;
7. Check if the parameters indicated by the area 1 in Fig. 13(a) are correct and match the embedded software; otherwise, correct them before continuing;
8. Start data acquisition by pressing the button indicated in area 2 in Fig. 13(a);
9. Data must appear in the received data fields and GUI graphs, except for the bottom center graph. If data reception is not verified, check the previous steps and use a serial monitor on the computer to check whether the problem is related to LabVIEW;
10. If the software is working as intended, press the load cell to ensure its data is correct; otherwise, check the previous steps;
11. Before connecting the igniters to the drive board, check the integrity of the wires using a continuity test. Be careful, as this is an extremely dangerous step. Perform this check in a safe place, away from the engine nozzle;
12. With the previous step successful, activate the lever indicated in area 3 in Fig. 13(a), and a confirmation message will be displayed confirming to activate the charges: “Are you sure you want to trigger the explosive charge? The delay is set to X seconds”. Be careful; this action will start the propellant burning after the configured trigger delay indicated in area 1 in Fig. 13(a). Make sure the test area is safe and everyone wears protective equipment, especially ear protectors. It is recommended to perform a dry-run validation of the relay output before connecting the igniter cable to ensure proper system behavior;
13. If data has been correctly sent to the hardware via the serial interface, the box indicated in area 4 in Fig. 13(a) will display the number of bytes of the standard message. Otherwise, there was an error in the software, and igniters were not fired;
14. After the test is complete, press the button indicated in area 5 in Fig. 13(a) to stop data acquisition. Please note that this indicates that no new data will be collected from the hardware, but the computer may be processing any remaining data in the FIFO queue. Wait until the bottom center graph displays all collected data throughout the test;
15. Turn off the system and disconnect all sensors and the serial cable.

## 7 Validation and characterization

Three distinct tests are depicted to demonstrate and characterize the DAQ hardware and its operation. First, the work presents the instrument’s noise analysis and compares it with the previous system that the Vector II group used before building the DAQ system. This system used a combination of a commercial JY-S60 amplification module and a NI USB-6221 multifunction DAQ. The first is a load cell transmitter amplifier, and the latter is a commercial data acquisition system from National Instruments.

Next, the calibration procedure results described in Section 6.3 are discussed. Lastly, the work depicts results obtained in actual engine tests. Notably, once more, some results are compared to the previous combination using the JY-S60 amplifier and the National Instruments DAQ.

### 7.1 Noise characterization

Before the experimental analysis, a theoretical evaluation of the expected system’s noise is shown. Table 4 displays the noise spectral density of the most critical components used in the hardware and its value at 100 Hz, the cutoff frequency of the first-order filters. Notably, the ADC data acquisition is 1000 Hz; consequently, there will be aliasing and higher frequency noise in the system, and the value in Table 4 is ideal and unattainable to be true in an experimental situation.

Nonetheless, the PGA308 is likely responsible for the system’s main noise, given that its estimated value is approximately 150μV, greater than the sum of all other components. For comparison purposes, the commercial hardware previously used in the Vector II Project, with the JY-S60 module and the NI USB-6221 board, only had available information about the National Instruments subsystem, whose noise is estimated at 244μVrms. The theoretical analysis shows that dedicated hardware may perform similarly to the more expensive combination of the commercial solution.

To experimentally compare both systems, a low-pass filter with a cutoff frequency of 100 Hz was added at the input of the NI USB-6221 ADC channel. Furthermore, two conditions were assessed: first, with a small standard weight placed in the load cell with very steady conditions, and second, with a Wheatstone bridge with precision resistors (0.1% 10-PPM resistors) simulating the load cell resistance.

**Table 4:** Theoretical noise analysis of the hardware components.

Component	Noise density	Noise at 100 Hz
PGA308	$50\times \text{Gain}\;\text{nV}/\sqrt{\text{Hz}}$	150 μV
SDADC	$\frac{V_{\mathrm{ref}}}{2^{\mathrm{ENOB}}\times \sqrt{12\times BW}}\;\text{V}/\sqrt{\text{Hz}}$	13.21μV
TPS7A49	–	≅20μV
REF3450	$1250\;\text{nV}/\sqrt{\text{Hz}}$	12.5μV
REF3430	$750\;\text{nV}/\sqrt{\text{Hz}}$	7.5μV

**Fig. 14:**
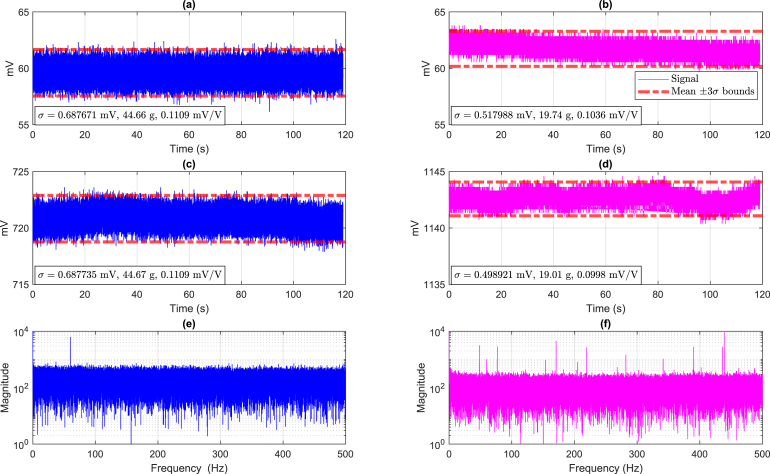
Noise Characterization Results. (a) Static load noise with the custom-built DAQ and (b) the commercial DAQ system. (c) External Wheatstone bridge noise with the custom-built DAQ and (d) the commercial DAQ system. (e) Static noise test Fast Fourier transform with for the custom-built DAQ and (f) the commercial DAQ system.

The test results can be seen in Fig. 14. It is worth noting that the difference in average output level between the graphs is due to the custom DAQ having a 3V input range, while the commercial system operates at 5V. The measured standard deviations are shown in Table 5, expressed in both output voltage (mV) and equivalent force (grams).

For a better comparison independent of supply voltage, Table 6 presents the noise characterization results normalized by the excitation voltage applied to the load cell in each system. The custom-built DAQ supplies approximately 6.2 V to the load cell, while the commercial DAQ uses a standard 5 V excitation. This difference directly affects the raw output signal amplitude and noise magnitude in millivolts.

**Table 5:** Standard deviation in noise characterization tests.

Test condition	Custom-built DAQ	Commercial DAQ solution
Static load	0.687671mV (44.66g)	0.517988mV (11.74g)
External Wheatstone bridge	0.687735mV (44.67g)	0.498921mV (19.01g)

When normalizing the standard deviation by the excitation voltage (expressed in mV/V), both systems demonstrate very similar intrinsic noise per unit of supply voltage, with the custom DAQ at approximately 0.1109mV/V and the commercial DAQ ranging between 0.0998 and 0.1036mV/V. This data shows that, despite having a slightly higher standard deviation absolute noise (about 180μV, equivalent to 25g in force), taking the excitation voltage into account, the custom DAQ normalized noise is only slightly higher (approximately 10%), demonstrating that the custom built DAQ has a comparable noise performance to the more expensive commercial counterpart.

**Table 6:** Normalized standard deviation in noise characterization tests.

Test condition	Custom-built DAQ	Commercial DAQ solution
Static load	0.1109mV/V	0.1036mV/V
External Wheatstone bridge	0.1109mV/V	0.0998mV/V

It is also essential to highlight a fundamental characteristic of the project over the commercial solution: its considerably lower cost. Although the commercial system presents a minor influence of noise on its measurements, its price is more than twenty times higher than that of the developed DAQ. Additionally, the noise characterization of 687μV of the custom-built hardware, corresponding to 15 Least Significant Bits (LSBs), is suitable for rocket engine testing. This value also aligns with the measurements performed for the known loads during the calibration process, whose standard deviations were around 700μV. Another important observation is the stability of the standard deviation across different test conditions for both systems, indicating the low contribution of noise from the load cell and the shielded cable.

In contrast to the theoretical data expected for 100 Hz, the experimental result displays higher noise values, as anticipated. Again, the first-order filter with a cutoff frequency of 100 Hz will not be ideal for the 1000 Hz acquisition rate from both DAQs. Still, the order of magnitude in both cases was comparable to the theoretical value. Although additional noise reduction could be attained with improved hardware design and filtering techniques, the results obtained are adequate for the operational requirements of the test bench.

Lastly, an analysis of the noises in the frequency domain was performed using the Fast Fourier Transform (FFT) and the data collected for static load noise, which encompasses all components during a real test condition. Fig. 14 presents the spectral analysis for the developed DAQ and the previous commercial system. For the custom-built DAQ, a steady noise band across the spectrum is evident, with spikes at 0 and 60 Hz. In contrast, the commercial system presents a smaller amplitude throughout the spectrum but with spikes in numerous frequencies.

These results demonstrate the effectiveness of the designed hardware and the custom-built DAQ results, as well as efficient techniques for routing printed circuit boards and dimensioning the different analog filters for the designed DAQ. The noise observed at 60 Hz is likely due to the influence of the power grid, whose higher frequency harmonics were attenuated both by the presence of the filters and by the transformers that supply the system. Even so, it is possible to filter the influence of the power grid by implementing new analog filters or even a digital Notch filter.

### 7.2 System calibration results

**Fig. 15:**
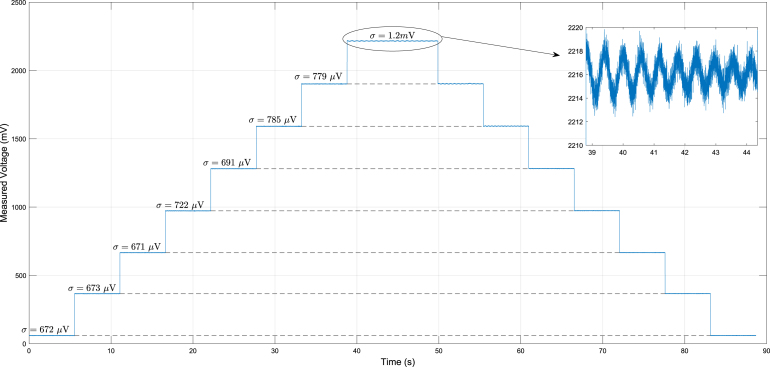
Load Cell Calibration results.

The manual calibration procedure described in Section 6.3 was performed using a set of seven INMETRO (Brazilian National Institute of Metrology, Standardization and Industrial Quality)-certified weights of 20 kg each. Fig. 15 depicts the data collected by the DAQ in millivolts and the standard deviation extracted from each weight, disregarding the measurements acquired during the transient oscillatory period caused by stacking the loads. The zoomed-in region also depicts that, in addition to the noise measurement, mechanical oscillations arise from the physical instability of the stacked weights, which introduces system instability and increases as the load stack grows. These oscillations become more pronounced at higher weight values, and would require additional time for the weight tower to stabilize indicating that an automated calibration system would be more suitable. Nonetheless, at the desired accuracy level, these oscillations have shown minimal impact on the calibration’s linear equation.

To further evaluate the system’s performance, measurements were also conducted during both the loading and unloading of the weights to evaluate potential hysteresis effects. The standard deviation values remained consistent in both cases. However, during the unloading process, oscillations tend to increase at certain weight levels due to manual handling, which may slightly affect short-term stability but does not compromise the overall calibration accuracy. Furthermore, as shown by the dashed lines in Fig. 15, the measured levels during loading and unloading closely correspond, indicating the absence of hysteresis.

Still, the standard deviation for each weight level was about 700μV, not far from the noise observed in the static test in the previous section, apart from the last weight level, which exhibited a discrepant deviation due to the greater influence of mechanical oscillations. The linear regression relating the ADC input voltage (or the PGA output one) and the force applied led to the linear relationship in (6). Lastly, it is essential to note that the wooden support for the weights is accounted for, which is why the 0 kg measurement has a residual mass measurement.

(6) $$F=636.9214\times V_{\text{ADC}}-19.7935,$$

where $V_{\text{ADC}}$ is the input voltage to the Sigma-Delta analog-to-digital converter (in volts), and F is the resulting force (in newtons).

### 7.3 Rocket engine tests

**Fig. 16:**
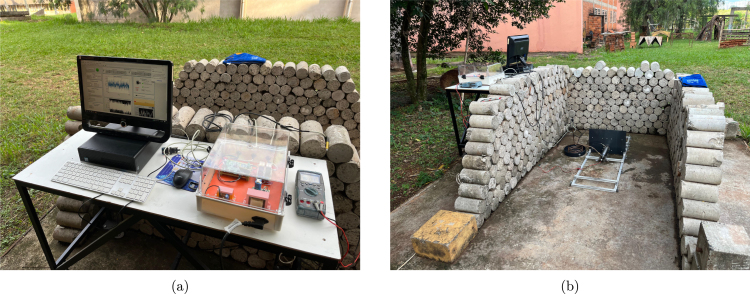
Test bench infrastructure for the Vector II project. (a) Hardware close-up View. (b) Physical structure.

Finally, the data acquisition system is validated in an actual test of an engine burn using the Vector II Project infrastructure, displayed in Fig. 16. The first subfigure has the DAQ hardware and the GUI, and the second details the physical infrastructure and the sensors, which consist of a system for fixing the engine and protective masonry.

**Fig. 17:**
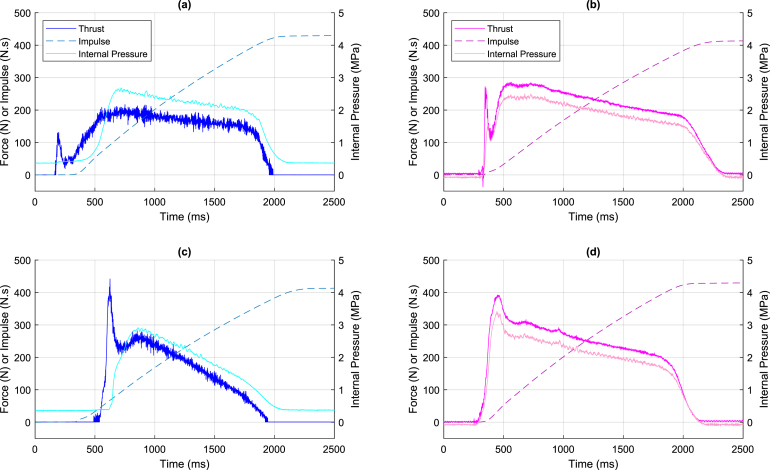
Experimental test acquired data. (a) Test with the custom-built DAQ and Propellant P1. (b) Test with the commercial DAQ system and Propellant P2. (c) Test with the custom-built DAQ and Propellant P3. (d) Test with the commercial DAQ system and Propellant P4.

Four sets of results are presented, two obtained with the custom-built DAQ and the others with the combination of the commercial solution using the JY-S60 amplification module and the NI USB-6221 DAQ. It is important to note that the propellant composition and ignition methods are different in all tests. The goal is to depict the success in extracting the load cell and pressure sensor measurements during the engine burn to obtain the thrust and impulse of the propellant and ignition combination. Fig. 17 depicts these results, where subfigures (a) and (c) are those using the custom-built DAQ and (b) and (d) using the commercial one.

Each respective graph displays the thrust and internal pressure measurements and the impulse curve obtained by integrating the thrust data over time. Comparing various propellants and both data acquisition systems allows us to ratify the effectiveness of the custom-built DAQ. It is possible to verify that the four thrust and pressure curves have a trapezoidal shape, which shows that all tests had an adequate burn. However, due to differences in the composition of each fuel, each data curve presents unique characteristics. Additionally, one should note that the oscillations in the graphs are not noise-related but rather due to the inherent behavior of propellant burning that causes oscillations in the engine and, consequently, vibrations in the load cell. The future addition of an inertial sensor, i.e., an accelerometer, could provide further insight into the matter.

### 7.4 Conclusions

These results validate the custom-built DAQ operation and performance. It combines the same functionalities of the previous hardware, utilizing the JY-S60 amplification module and the NI USB-6221 DAQ at a considerably lower cost. Furthermore, it has safety features tailored specifically to an engine test bench, which are unavailable in commercial DAQ systems.

Lastly, the following capabilities and limitations of the hardware are summarized based on the project development, characterization, and field performance:

• The custom-built hardware provides a much cheaper solution to the test bench than the commercial one, which combines a load cell amplifier with the National Instruments DAQ;
• It has a simple operation, allowing users to focus on safety procedures inherent in experiments with rocket engines, minimizing involved risks;
• It contains internal protection circuits to limit voltages in case of drawbacks and an engine’s catastrophic failure during testing, in addition to an isolated serial communication interface using the RS-232 signaling;
• Intuitive operating Graphical User Interface to facilitate the implementation of experimental tests;
• High versatility and modularity for later expansions, such as sensors to measure other physical matters during the engine burn, such as temperature and vibrations;
• Plug and Play hardware with no adaptations necessary for use; it is only required to program the PGA308 with the characteristics of the load cell to be used;
• The design and fabrication process is straightforward, allowing easy adaptation and replication using the provided design files;
• Future versions could support USB communication, in addition to the RS-232 interface, which would make usage simpler for the end user;
• A graphical interface directly linked to the hardware and developing an SD card storage would eliminate the need for a dedicated computer;
• Using two relays connected serially for each igniter would increase the system’s safety, preventing accidental ignitions due to relay malfunctioning.

## CRediT authorship contribution statement

**Nathan Andreani Netzel:** Writing – original draft, Validation, Software, Methodology, Investigation, Data curation, Conceptualization. **Daniel Strufaldi Batista:** Writing – review & editing, Validation, Supervision, Conceptualization. **Francisco Granziera Jr.:** Writing – review & editing, Validation, Conceptualization. **Marcelo Carvalho Tosin:** Writing – review & editing, Validation, Supervision, Resources, Project administration, Methodology, Investigation, Funding acquisition, Conceptualization.

## Declaration of competing interest

The authors declare that they have no known competing financial interests or personal relationships that could have appeared to influence the work reported in this paper.
